# Computational Studies on Holey TMC_6_ (TM = Mo and W) Membranes for H_2_ Purification

**DOI:** 10.3390/membranes12070709

**Published:** 2022-07-14

**Authors:** Juan Xie, Cai Ning, Qinqin Liu, Zhongti Sun, Juan Yang, Huilong Dong

**Affiliations:** 1 School of Materials Science and Engineering, Jiangsu University, Zhenjiang 212013, China; xiejuan@cslg.edu.cn (J.X.); qqliu@ujs.edu.cn (Q.L.); 2 School of Materials Engineering, Changshu Institute of Technology, Changshu 215500, China; 3 School of Physics, Southeast University, Nanjing 211189, China; caining54@njust.edu.cn

**Keywords:** carbon-based membrane, hydrogen purification, first-principle calculations, selectivity

## Abstract

The purification of hydrogen (H_2_) has been a vital step in H_2_ production processes such as steam–methane reforming. By first-principle calculations, we revealed the potential applications of holey TMC_6_ (TM = Mo and W) membranes in H_2_ purification. The adsorption and diffusion behaviors of five gas molecules (including H_2_, N_2_, CO, CO_2_, and CH_4_) were compared on TMC_6_ membranes with different phases. Though the studied gas molecules show weak physisorption on the TMC_6_ membranes, the smaller pore size makes the gas molecules much more difficult to permeate into h-TMC_6_ rather than into s-TMC_6_. With suitable pore sizes, the s-TMC_6_ structures not only show an extremely low diffusion barrier (around 0.1 eV) and acceptable permeance capability for the H_2_ but also exhibit considerably high selectivity for both H_2_/CH_4_ and H_2_/CO_2_ (>10^15^), especially under relatively low temperature (150–250 K). Moreover, classical molecular dynamics simulations on the permeation process of a H_2_, CO_2_, and CH_4_ mixture also validated that s-TMC_6_ could effectively separate H_2_ from the gas mixture. Hence, the s-MoC_6_ and s-WC_6_ are predicted to be qualified H_2_ purification membranes, especially below room temperature.

## 1. Introduction

With the increasing environmental pollution issues and global energy crisis, more and more attention has been paid to green energy resources, particularly hydrogen (H_2_) energy [1,2]. Though lots of methods have been developed, steam–methane reforming is still the main approach for industrial production of H_2_ [3]. During the steam–methane reforming process, however, the mixture composed of H_2_, CO_2_, and CH_4_ is inevitable, which renders the purification of H_2_ highly crucial in hydrogen production. The membrane separation technology is one of the most widely accepted methods for H_2_ separation and purification [4]. Among the reported membranes, two-dimensional (2D) carbon-based membranes have been extensively studied due to their distinct advantages, e.g., low energy consumption and good cyclicity through physical interactions [5,6,7].

Material design by computational methods has been an effective tool to achieve novel 2D carbon-based ultrathin membranes for H_2_ separation and purification, especially for the ones with intrinsic pores. As one of the most well-known representatives, the porous graphitic carbon nitride (g-C_3_N_4_) monolayer has received a lot of attention for its potential as an effective gas separation membrane. Under room temperature, g-C_3_N_4_ could exhibit extremely high theoretical selectivity for H_2_/CH_4_ in the order of 10^46^ [8]. Moreover, further theoretical simulations indicated that g-C_3_N_4_ is also capable in helium (He) purification from both natural gas and noble gas molecules [9]. With the help of theoretical simulations, the porous C_2_N monolayer was reported to be suitable for He separation from other gases (Ne, CH_4_, CO_2_, etc.) [10]. Wang et al. investigated the diffusion properties of He, Ne, CO_2_, Ar, N_2_, CO, and CH_4_ through a porous monolayer covalent triazine-based framework (CTF) membrane. Calculation results demonstrate that the selectivity for He and H_2_ against common gas molecules (such as CO_2_, N_2_, CO, and CH_4_) is highly promising for practical applications [11]. Meng et al. theoretically explored the structural and mechanical properties of metal-free fused-ring polyphthalocyanine (H_2_PPc) and halogenated H_2_PPc (F-H_2_PPc and Cl-H_2_PPc) membranes. It was found that fluorination and chlorination can effectively tune the permeable pores. Particularly, F-H_2_PPc is fascinating as a separation membrane for H_2_ purification [12]. Recently, a series of 2D γ-C_4_X (X = O, S, or Se) membranes with intrinsic pores were theoretically designed, among which γ-C_4_O shows both low diffusion barriers (0.35 eV) and high permeance (5.0 × 10^−7^ mol m^−2^ s^−1^ Pa^−1^) for H_2_. Moreover, γ-C_4_O is highly promising as a H_2_ purification membrane from the H_2_/CH_4_ mixture with a selectivity of about 10^19^ [13]. The existing studies indicate that there are abundant possibilities for carbon-based ultrathin membranes with intrinsic pores. Therefore, carbon-based membranes with different pore sizes and termination on the pore edges are indispensable for H_2_ purification.

Besides the ones entirely composed of non-metal elements, 2D membranes composed of carbon and metal atoms may also play a vital role as gas separation membranes due to the incorporation of metal atoms. In the pioneering work by Li et al., a novel 2D transition metal carbide (h-TMC_6_, TM = Mo, W) structure was theoretically designed [14]. It was found that the crystal structure of h-TMC_6_ belongs to the hexagonal Kagome lattice. The stability of h-TMC_6_ was confirmed by molecular dynamics simulations and phonon spectra calculations. Later, Liu et al. reported other transition-metal carbides with the same composition of TMC_6_ (TM = Mo, W) but a tetragonal lattice [15], therefore being named s-TMC_6_. In general, the TMC_6_ monolayers show triple atomic layer structures with Mo/W atomic layers sandwiched between two carbon atomic layers, with TM atoms coordinated with six nearest neighboring C atoms. More importantly, in both h-TMC_6_ and s-TMC_6_, there are intrinsic pores surrounded by the TM atoms and carbon atoms, whose sizes are mainly determined by the lattice structures.

In this work, the capability of holey structures of TMC_6_ (M = Mo, W) membranes for H_2_ purification was theoretically explored, both square and hexagonal phases. By comparing the pore size and separation performance against H_2_ and other gas molecules (N_2_, CO, CO_2_, CH_4_), it was found that s-TMC_6_ is more promising for H_2_ purification, especially from H_2_/(CO_2_, CH_4_) mixtures below room temperature (150–250 K). Our work not only predicts the potential applications of the TMC_6_ membranes but also recommends the novel membrane materials for H_2_ purification under low temperature.

## 2. Computational Methods

The Vienna *ab initio* simulation package (VASP) [16,17] was used for the first-principle calculations with plane-wave basis set and the projector augmented-wave (PAW) [18] method. The Perdew–Burke–Ernzerhof (PBE) [19] functional was adopted, with a cutoff energy of 500 eV. The structural relaxations were considered to be converged until the change in total energy reaches 10^−5^ eV and the residual force per atom reaches 0.02 eV Å^−1^. To avoid the interaction between the neighboring periodic images, a vacuum slab around 2 nm was applied for all the structures. The Brillouin zone was sampled with Monkhorst–Pack [20] *k*-point grids of 11 × 11 × 1 and 5 × 5 × 1 for unit cell of h-TMC_6_ and s-TMC_6_, respectively. Furthermore, 4 × 4 × 1 *k*-point grids were adopted for the 3 × 3 supercell of h-TMC_6_, while 3 × 3 × 1 *k*-point grids were adopted for the 2 × 2 supercell of s-TMC_6_. For accurate description of weak van der Waals (vdW) interactions, Grimme’s dispersion correction (DFT–D3) was included during the adsorption-related calculations [21]. The climbing image nudged elastic band (CI-NEB) method [22] was used to search the minimum energy pathway (MEP) and confirm the transition state (*TS*) during the diffusion of gas molecules. The diffusion barriers (*E_b_*) were calculated to evaluate the capability of gas molecules passing through the intrinsic pores in the TMC_6_ membranes by the following definition:

(1) $$E_{b}=E_{TS}-E_{IS}$$

where the *E_TS_* and *E_IS_* denote the energy of transition state (*TS*) and initial state (*IS*), respectively. Additionally, the adsorption energies (*E_ad_*) of gas molecules on 2D TMC_6_ were calculated by

(2) $$E_{ad}=E_{mol*}-E_{mol}-E_{*}$$

where $E_{mol*}$, $E_{mol}$, and $E_{*}$ stand for the energy of adsorption system, isolated molecule, and 2D TMC_6_, respectively. Herein, a negative *E_ad_* value means that the adsorption is favorable as exothermic process.

The classical molecular dynamics (MD) simulations on permeation process of H_2_, CO_2_, and CH_4_ mixture were implemented by Forcite module available in the Materials Studio software package. Gas molecules were interspersed between the TMC_6_ membranes, and the initial condition was 250 K with a total simulation time of 5000 ps. The NVT ensemble and universal force field [23] were employed during the simulation with a time step of 1 fs.

## 3. Results and Discussion

**Figure 1:**
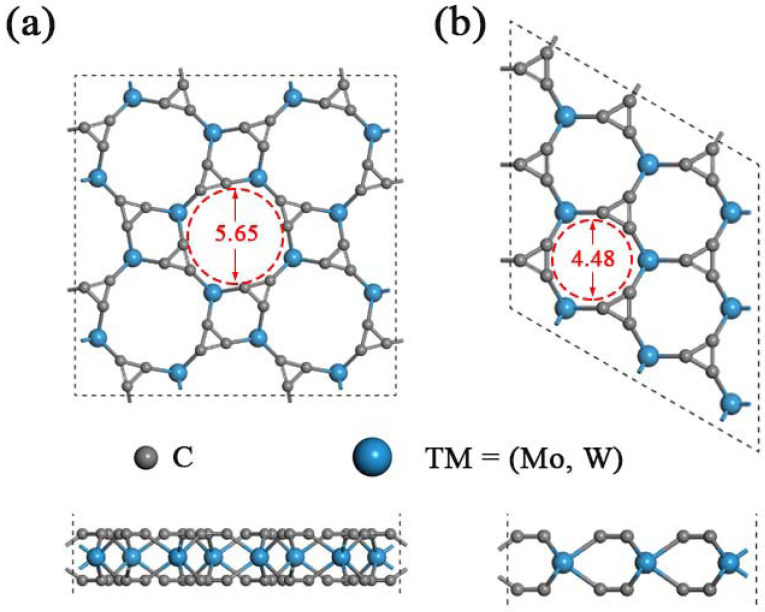
Schematic illustrations of TMC_6_ (top view and side view) supercells: (**a**) Square transition-metal carbides s-TMC_6_; (**b**) hexagonal transition-metal carbides h-TMC_6_. The gray and blue balls represent C and TM atoms, respectively.

As predicted in previous work, the single-layered TMC_6_ (M = Mo, W) includes two different phases with holey structure, namely, the square phase (s-TMC_6_) and the hexagonal phase (h-TMC_6_). s-TMC_6_ shows *P4/mbm* symmetry with a lattice constant of 8.541 and 8.543 Å for s-MoC_6_ and s-WC_6_, respectively. There are 4 TM atoms and 24 carbon atoms in the unit cell of s-TMC_6_. Due to the extremely close lattice parameters, s-MoC_6_ and s-WC_6_ exhibit intrinsic pores with the same diameter of about 5.65 Å, which were obtained by directly measuring from the optimized atomic positions of carbon in the edge of pores. The intrinsic pores are composed of four TM atoms and eight C atoms, as displayed in Figure 1a. Differently, the h-TMC_6_ exhibits *P*$\bar{6}$*m2* symmetry with the lattice constant of 4.381 and 4.383 Å for h-MoC_6_ and h-WC_6_, respectively. The unit cell of h-TMC_6_ contains one TM atom and six carbon atoms. Due to the more compact atomic configurations of h-TMC_6_, their intrinsic pores have smaller sizes when compared with s-TMC_6_. As indicated in Figure 1b, the pores in h-TMC_6_ are composed of three TM atoms and six C atoms, whose diameters are about 4.48 Å.

**Figure 2:**
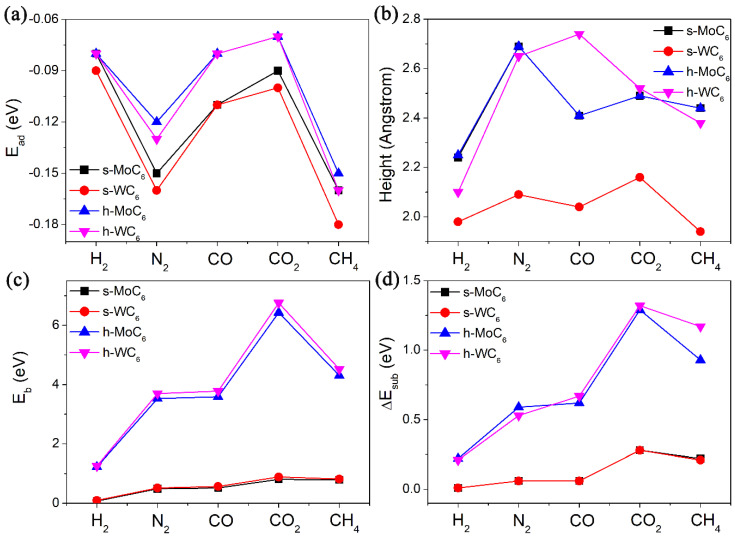
(**a**) The adsorption energies (*E_ad_*, eV), (**b**) equilibrium adsorption heights (h, Å), (**c**) diffusion barriers for the studied gases (H_2_, N_2_, CO, CO_2_, and CH_4_) passing through the intrinsic pore (*E_b_*, eV) of 2D TMC_6_, and (**d**) energy differences (Δ*E_sub_*, eV) of the TMC_6_ membranes between free-standing state and TS without adsorbates.

After the confirmation of pore structures in the 2D TMC_6_, we then tested their performance in gas adsorption and diffusion. In this work, five different kinds of gas molecules (containing H_2_, N_2_, CO, CO_2_, and CH_4_) were tested, which are the main components of the gas mixture in the steam–methane reforming process. All the adsorption configurations of gas molecules were fully relaxed. The adsorption energies (*E_ad_*), equilibrium adsorption heights (h), and diffusion barriers (*E_b_*) are systematically summarized in Table 1. In the equilibrium configurations, the adsorption heights between the gas molecules and substrate are mostly in the range of 2.0–2.5 Å, along with adsorption energies within −0.18 eV, as shown in Figure 2a,b. The large adsorption heights and weak adsorption strength evidently indicate physisorption through van der Waals interaction between the gas molecules and TMC_6_ substrates. Generally speaking, the *E_ad_* of the same gas molecule on MoC_6_ and WC_6_ is almost the same, implying the negligible influence of TM atoms on the gas adsorption. Meanwhile, the *E_ad_* values of a gas molecule on s-TMC_6_ will be slightly smaller than those on h-TMC_6_. However, it is interesting to see that the diffusion of the same molecule on h-TMC_6_ will be much more difficult than that on s-TMC_6_ due to the much greater *E_b_* values (see Figure 2c). Another finding is that for s-MoC_6_ and s-WC_6_, due to the relatively small *E_b_* values, the difference between *E_b_* of the same gas molecule on them is also very small (<0.1 eV). For h-MoC_6_ and h-WC_6_, a significant difference between *E_b_* of the same gas molecule on them arises (up to 0.33 eV). Therefore, though the gas molecules have close adsorption interaction on the TMC_6_ membranes, their diffusion performance is highly distinguished.

**Figure 3:**
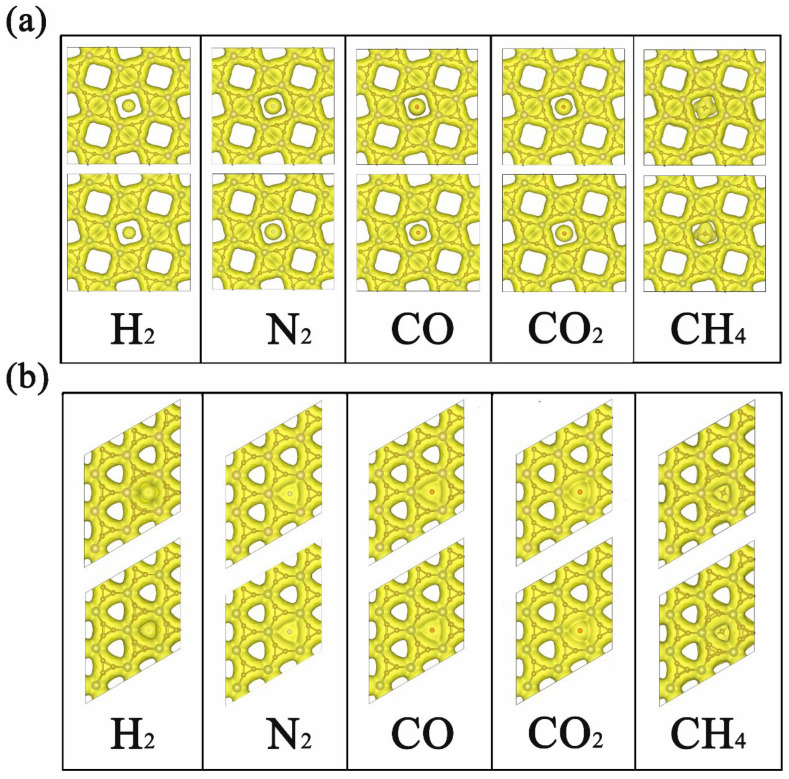
Electron density isosurfaces of H_2_, N_2_, CO, CO_2_, and CH_4_ on (**a**) s-TMC_6_ and (**b**) h-TMC_6_ at their corresponding transition states (TSs). The upper panels mark MoC_6_, while the lower panels denote WC_6_. The isosurface value is 0.015 e Å^−3^.

After the comparison of adsorption and diffusion performance of different gas molecules on 2D TMC_6_, we then turned to the microscopic insights. As displayed in Figure 3, the electron density isosurfaces of H_2_, N_2_, CO, CO_2_, and CH_4_ at their corresponding transition states are provided with the same isosurface value of 0.015 e Å^−3^. For s-TMC_6_, it is evident that the smaller pores are fully occupied by the electron density isosurfaces, and the unoccupied parts only appear in larger pores with square shapes. Meanwhile, for h-TMC_6_, the triangle shapes appear in the pores as unoccupied parts, whose area is significantly smaller than that in s-TMC_6_. As we can see, under the transition states, there is significant overlapping between the electron density distribution of h-TMC_6_ and gas molecules, while the overlapping between s-TMC_6_ and gas molecules is lower, especially for H_2_, N_2_, CO, and CO_2_. The overlapping of electron density distribution will cause significant electrostatic interactions. The electrostatic interaction plays a leading part during the permeation process because higher overlapping of electron density distribution corresponds to larger E_b_ values. In this work, the electrostatic interaction mainly originates from the different pore sizes of 2D TMC_6_. As a useful tool to quantitatively understand the selectivity of TMC_6_ against different gases, a comparison between the measured diameters of the cross section in the van der Waals (vdW) surface (D_c_) of different gas molecules and the pore size of unoccupied vdW surface in 2D TMC_6_ was also performed. As measured, the unoccupied vdW diameters of pore in s-MoC_6_ and s-WC_6_ are 2.29 and 2.32 Å, significantly higher than those of g-C_3_N_4_ (about 1.70 Å) [8] which results in significantly smaller E_b_ values for gas molecules permeating g-C_3_N_4_ when compared with s-TMC_6_. As reported, the D_c_ of H_2_, N_2_, CO, CO_2_, and CH_4_ is 2.44, 3.20, 3.46, 3.44, and 3.78 Å, respectively. Therefore, the pore size of s-TMC_6_ is very close to the D_c_ of H_2_ but much smaller than those of N_2_, CO, CO_2_, and CH_4_. This finding means that s-TMC_6_ will have considerable selectivity against H_2_, attributed to the suitable pore size. Notably, though the pore size of s-WC_6_ is slightly larger than that of s-MoC_6_, it causes higher E_b_ for the same gas molecule. Hence, the metal species has relatively less influence on the diffusion of gas molecules, but it is not negligible. In the meantime, the unoccupied vdW diameters of pore in h-TMC_6_ are 0, which explains why h-TMC_6_ exhibits very high diffusion barriers for all the studied gas molecules.

In addition to the comparison between pore sizes, the steric deformation of 2D TMC_6_ caused by electrostatic repulsion was also checked. We measured the main bond lengths of different molecules in isolated state (*l*_1_) and TS (*l*_2_), that is, H-H bond length for H_2_, N-N bond length for N_2_, C-O bond length for CO and CO_2_, and C-H bond length for CH_4_, taking in consideration the non-polarized structures of CO_2_ and CH_4_. The comparison between *l*_1_ and *l*_2_ (seeing Table 1) indicates that the structural changes of the gas molecules under TS are negligible, except for CO and N_2_ on h-TMC_6_. Then, by removing the gas molecule in its TS structure and calculating the single-point energy (SPE) of the 2D TMC_6_, we can obtain the energy differences (Δ*E_sub_*) of the 2D TMC_6_ between free-standing state and TS without adsorbates, as listed in Table 1. It can be summarized that the Δ*E_sub_* is directly proportional to the corresponding *E_b_*. For H_2_, N_2_, and CO, the electrostatic repulsion takes the proportion of 10–18% in the *E_b_*, and the proportion increases to 20–30% for CO_2_ and CH_4_. It is revealed that CO_2_ and CH_4_ have more significant electrostatic repulsion with the pore edge of 2D-TMC_6_ when compared with other smaller gas molecules. Moreover, as indicated by Figure 2d, the Δ*E_sub_* in CO_2_ involved TS is obviously larger than that in the corresponding CH_4_ involved TS, implying stronger electrostatic repulsion of CO_2_ to the substrate. This finding explains the origin of the high *E_b_* values of CO_2_ permeating into the pores of 2D TMC_6_.

To evaluate the gas separation performance quantitatively, we calculated the permeability and selectivity based on diffusion energy barriers. For the permeability, gas kinetic theory was employed under the ideal gas approximation. Herein, the permeance (*P*, in mol m^−2^ s^−1^ Pa^−1^) of the penetrated gases is determined by [12]

(3) $$\;\;\;\;P=\frac{A_{p}N\int_{v_{b}}^{\infty }f\left(v\right)dv}{A_{m}\Delta pN_{A}}=\frac{A_{p}}{A_{m}}\frac{p}{\Delta p}\frac{1}{N_{A}\sqrt{2\pi mk_{B}T}}\int_{v_{b}}^{\infty }f\left(v\right)dv$$

In Equation (1), *A*_p_ denotes the area of the pores, and the total number of collisions per unit time per area (*N*) is described as $\;N=\frac{p}{2\pi mk_{B}T}$, where *p*, *m*, *k_B_*, and *T* stand for pressure, the mass of the molecule, the Boltzmann constant, and temperature, respectively. Hence, *A*_p_*N* could be viewed as the number of molecules that collide with the pore area per unit time. The portion of molecules with a speed large enough to overcome the diffusion barrier through the pore (i.e.,$\;v>v_{b}=\sqrt{\frac{2E_{b}}{m}}$) is counted as the penetrant portion. *A*_m_, Δp, and f(v) represent the total area of the membrane, the pressure difference (absolute value) between the two sides of the membrane, and the Maxwell velocity distribution, respectively. It is worth noting that *A*_m_ is explicit for a 2D membrane, while *A*_p_ is related to the pore shapes and the effective radii (*R*_eff_) of the atoms at the pore rim. Here, *R*_eff_ is calculated as$\;R_{\mathrm{eff}}=R_{\mathrm{vdw}}/\sqrt{2}$, where *R*_vdW_ denotes the vdW radius. The feed pressure and the pressure difference are $p=3\times 10^{5}$ Pa and $\Delta p=10^{5}$ Pa as provided in previous work [24].

**Figure 4:**
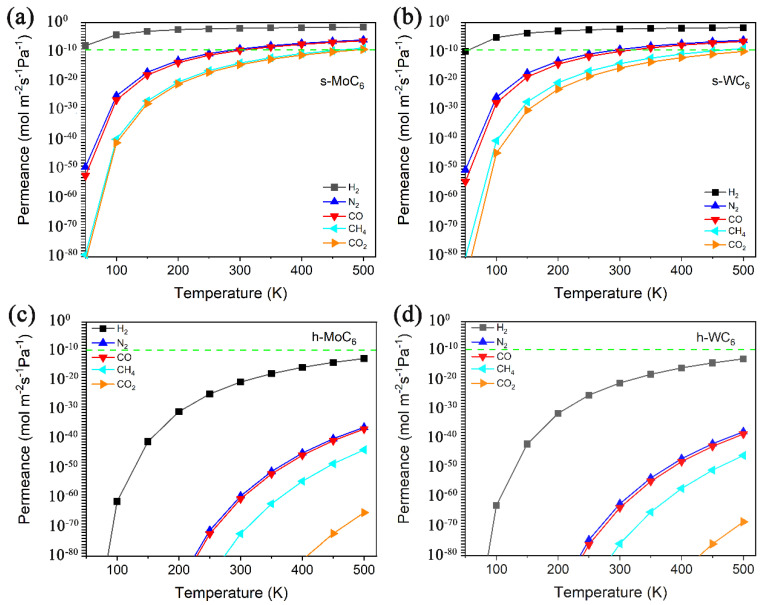
Permeance versus temperature for H_2_, N_2_, CO, CO_2_, and CH_4_ passing through the intrinsic pore of (**a**) s-MoC_6_, (**b**) s-WC_6_, (**c**) h-MoC_6_, and (**d**) h-WC_6_.

The calculated permeance vs. temperature for H_2_, N_2_, CO, CO_2_, and CH_4_ passing through the intrinsic pore of s-TMC_6_ and h-TMC_6_ are displayed in Figure 4. The green dashed line indicates the industrially acceptable permeance capability for gas separation (green dashed line, 6.7 × 10^−9^ mol m^−2^ s^−1^ Pa^−1^). Over the temperature range of 100–500 K, the permeance values for each gas molecule through s-TMC_6_ are evidently larger than those through h-TMC_6_. s-TMC_6_ shows good permeance capability against hydrogen, while h-TMC_6_ has low permeance capability for the different gas molecules. Specifically, in the temperature range of 100–300 K, the permeance values of H_2_ through s-TMC_6_ are always higher than the industrially acceptable one for gas separation. Meanwhile, the permeance values of CO_2_ and CH_4_ are always lower than the standard. For N_2_ and CO, the permeance values will not be higher than the standard until the temperature is higher than 300 K. It is suggested that s-TMC_6_ could be a potential H_2_ purification membrane to separate H_2_ from a mixture composed of N_2_, CO, CO_2_, or CH_4_ below room temperature (100–300 K).

It is well accepted that the performance of a separation membrane is characterized by both the permeance capability and selectivity. Herein, the selectivity between two gas species is defined as the ratio of the diffusion rates, *S*_gas-1/gas-2_ = *A*_gas-1_/*A*_gas-2_, which comes from the Arrhenius equation:

(4) $$A=A_{0}exp\left(-E_{b}/k_{B}T\right)$$

**Figure 5:**
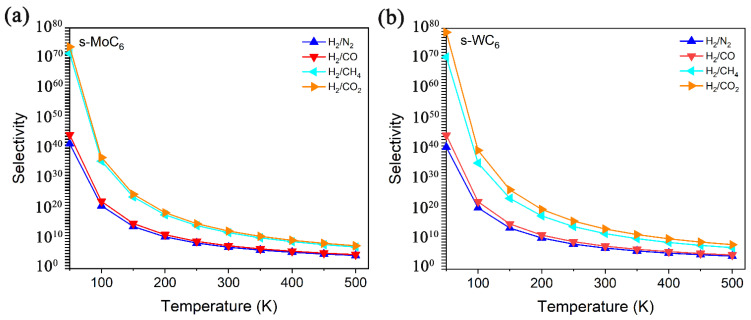
Selectivity versus temperature for H_2_/N_2_, H_2_/CO, H_2_/CH_4_, and H_2_/CO_2_ separation by (**a**) s-MoC_6_, (**b**) s-WC_6_.

In Equation (2), the *A*_0_ means the diffusion prefactor that can be taken as the same value for all gases (10^11^ s^−1^). Based on the definition, the selectivity gradually decreases with the increase in temperature. In Figure 5, the selectivity versus temperature for H_2_/N_2_, H_2_/CO, H_2_/CH_4_, and H_2_/CO_2_ separation by s-MoC_6_ and s-WC_6_ are illustrated. It is easy to find that the s-TMC_6_ membranes mainly exhibit excellent selectivity for H_2_/CH_4_ and H_2_/CO_2_ due to the great differences in E_b_ between H_2_ and CH_4_/CO_2_. Different from other carbon-based 2D membranes, under room temperature (300 K), the selectivity of s-TMC_6_ for H_2_ against other gas molecules is not ideal enough, with 10^11^ and 10^13^ for H_2_/CH_4_ and H_2_/CO_2_, respectively. It is suggested that s-TMC_6_ could be applied as H_2_ purification membranes under low temperatures (100–250 K), with the selectivity of H_2_/(CO_2_, CH_4_) larger than 10^15^. As indicated by the selectivity, s-WC_6_ possesses better H_2_ purification ability when compared with s-MoC_6_.

The convection–diffusion process is a problem in the field of fluid mechanics. Generally, the finite difference method (FDM) is a major method to treat with the convection–diffusion equation [25] which only applies to macroscopic systems. However, for microscopic systems such as the TMC_6_ membranes in this work, it is extremely difficult to perform quantitative calculations on convection and diffusion of gas flow. As a result, the MD simulations were widely adopted to visualize the time-dependence diffusion process of the molecules, as well as to assess some parameters such as gas diffusion coefficient and the permeated number of gas molecules [26,27,28,29]. Due to the lack of quantitative results, MD simulations are often applied to confirm the results of DFT calculations.

**Figure 6:**
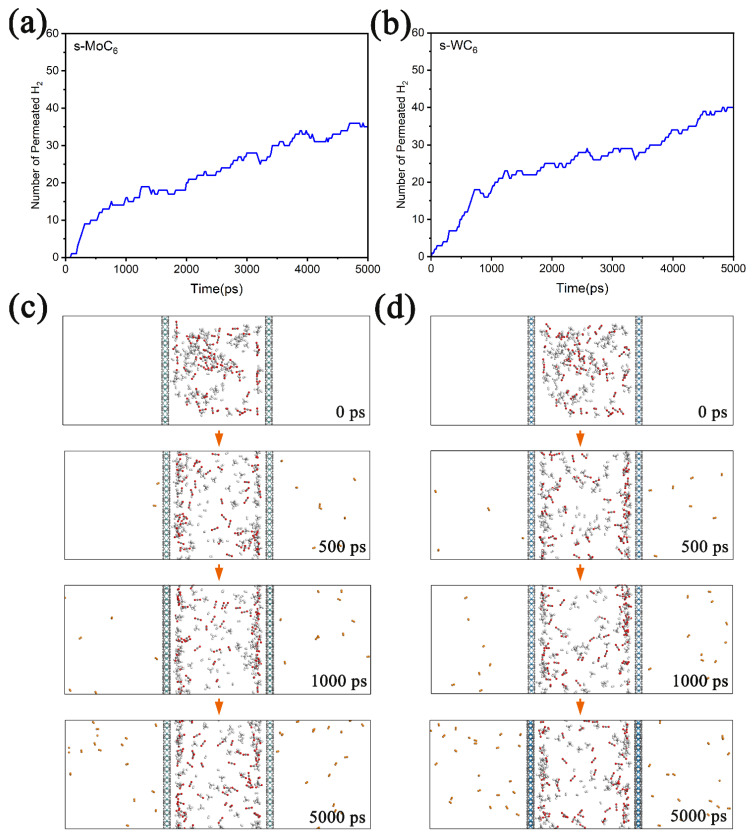
The number of permeated H_2_ molecules versus the simulation time for gas mixture in chamber composed of (**a**) s-MoC_6_ membranes and (**b**) s-WC_6_ membranes. Corresponding snapshots of the diffusion process (from 0 to 5000 ps) of gas mixture permeating through (**c**) s-MoC_6_ membranes and (**d**) s-WC_6_ membranes. The H_2_ molecules that diffuse outside are displayed by different color.

To better understand the transmembrane processes during gas separation, we performed classical MD simulations (at 250 K) on the permeation of gas mixture through the TMC_6_ membranes. During the MD simulations, a box of about 4 nm × 4 nm × 12 nm was employed; then, 60 H_2_, 60 CO_2_, and 60 CH_4_ molecules were randomly put into a chamber composed of two TMC_6_ membranes (the distance between the membranes was set as 4 nm). In Figure 6a,b, the number of permeated H_2_ molecules versus the simulation time for gas mixture in the chamber composed of s-TMC_6_ are depicted. It is found that after MD simulations of 5000 ps, there are 35 and 40 H_2_ molecules diffusing outside of the s-MoC_6_ and s-WC_6_ membranes, respectively. It is worth noting that the diffusion equilibrium was not achieved within 5000 ps; we can expect that after a long enough time, all the H_2_ molecules will diffuse through the s-TMC_6_ membranes into the product chamber. The snapshots of the gas mixture permeating through the s-TMC_6_ membranes at 0 ps, 500 ps, 1000 ps, and 5000 ps are given in Figure 6c,d. During the permeation process, the H_2_ molecules gradually migrate from the feed chamber to the product chamber. Moreover, none of CO_2_ or CH_4_ is found outside the membranes, clearly indicating that the s-TMC_6_ membranes could efficiently separate H_2_ molecules from the H_2_, CO_2_, and CH_4_ mixture. Therefore, the MD simulations could well simulate the transmembrane processes of H_2_ molecules, which greatly supports our first-principle calculation results.

In addition, it should be mentioned that we also performed the same MD simulations on h-TMC_6_. However, there are no gas molecules that run out from the chamber after 5000 ps at 250 K, indicating that h-TMC_6_ could not work as effective gas separation membranes.

## 4. Conclusions

In this work, the potential applications of holey TMC_6_ membranes in H_2_ purification were uncovered by comparative first-principle calculations. The adsorption and diffusion behaviors of five gas molecules (including H_2_, N_2_, CO, CO_2_, and CH_4_) were investigated on h-TMC_6_ and s-TMC_6_. All the studied gas molecules showed weak physisorption on the TMC_6_ membranes, but distinguishing diffusion barriers were obtained for different gas molecules across the pores of TMC_6_ membranes. The smaller pore size makes the gas molecules much more difficult to permeate into h-TMC_6_ rather than into s-TMC_6_. With suitable pore sizes, the s-TMC_6_ structures not only show an extremely low diffusion barrier and acceptable permeance for the H_2_ but also exhibit considerably high selectivity for H_2_/CH_4_ and H_2_/CO_2_, under relatively low temperature (150–250 K). Moreover, classical MD simulations on the permeation process also validated that the s-TMC_6_ could effectively separate H_2_ from the gas mixture composed of H_2_, CO_2_, and CH_4_. Therefore, s-MoC_6_ and s-WC_6_ are qualified as separation membranes for H_2_ purification from a gas mixture consisting of H_2_, CH_4_, and CO_2_ below room temperature.

## Figures and Tables

**Table 1:** The adsorption energies (*E_ad_*, eV), equilibrium adsorption heights (h, Å), diffusion barriers for the studied gases (H_2_, N_2_, CO, CO_2_, and CH_4_) passing through the intrinsic pore (*E_b_*, eV) of 2D TMC_6_, energy differences (Δ*E_sub_*, eV) of the TMC_6_ membranes between free-standing state and TS without adsorbates, the bond lengths (*l*_1_ and *l*_2_, Å) of the gas molecules with free-standing state and TS.

	Property	H_2_	N_2_	CO	CO_2_	CH_4_
s-MoC_6_	*E_ad_*	−0.08	−0.15	−0.11	−0.09	−0.16
	*h*	2.24	2.69	2.41	2.49	2.44
	*E_b_*	0.08	0.49	0.52	0.81	0.79
	Δ*E_sub_*	0.01	0.06	0.06	0.28	0.22
	*l*_1_	0.75	1.11	1.14	1.18	1.10
	*l*_2_	0.75	1.12	1.15	1.18	1.10
s-WC_6_	*E_ad_*	−0.09	−0.16	−0.11	−0.10	−0.18
	*h*	1.98	2.09	2.04	2.16	1.94
	*E_b_*	0.10	0.52	0.57	0.89	0.82
	Δ*E_sub_*	0.01	0.06	0.06	0.28	0.21
	*l*_1_	0.75	1.11	1.14	1.18	1.10
	*l*_2_	0.75	1.12	1.15	1.18	1.10
h-MoC_6_	*E_ad_*	−0.08	−0.12	−0.08	−0.07	−0.15
	*h*	2.25	2.69	2.41	2.49	2.44
	*E_b_*	1.23	3.53	3.59	6.43	4.31
	Δ*E_sub_*	0.22	0.59	0.62	1.29	0.93
	*l*_1_	0.75	1.11	1.14	1.18	1.10
	*l*_2_	0.74	1.15	1.18	1.18	1.10
h-WC_6_	*E_ad_*	−0.08	−0.13	−0.08	−0.07	−0.16
	*h*	2.10	2.65	2.74	2.52	2.38
	*E_b_*	1.26	3.70	3.78	6.76	4.52
	Δ*E_sub_*	0.21	0.53	0.67	1.32	1.17
	*l*_1_	0.75	1.11	1.14	1.18	1.10
	*l*_2_	0.74	1.15	1.18	1.19	1.10
